# Introduction of the HAM-Nat examination – applicants and students admitted to the Medical Faculty in 2012-2014

**DOI:** 10.3205/zma000995

**Published:** 2015-11-16

**Authors:** Katrin Werwick, Kirstin Winkler-Stuck, Wolfgang Hampe, Peggy Albrecht, Bernt-Peter Robra

**Affiliations:** 1 Otto-von-Guericke University Magdeburg, Medical Faculty, Office of the Dean of Studies, Magdeburg, Germany; 2 Hamburg-Eppendorf University Hospital, Centre for Experimental Medicine, Institute for Biochemistry and Molecular Cell Biology, Hamburg, Germany; 3 Otto-von-Guericke University Magdeburg, Medical Faculty, Institute for Social Medicine and Health Economics, Magdeburg, Germany

**Keywords:** Selection procedure, HAM-Nat, Study of Medicine, Student Survey, University Admission Procedure (AdH), Selection of Applicants to Study Medicine

## Abstract

**Background/aim:** In the 2012/13 winter semester, the Magdeburg Medical Faculty introduced a test of knowledge for the selection of applicants. The Hamburg Assessment Test for Medicine - Natural Sciences (HAM-Nat) comprises a multiple-choice test with questions on the aspects of biology, physics, chemistry and mathematics relevant to medicine, which was specifically developed for the selection of medicine applicants.

The aim is to study how the HAM-Nat influences student selection, the reasons why students decide to take the test as part of their application procedure and what expectations they have of their course of study.

**Methods: **The selection procedures applied at the university in 2011 (without HAM-Nat) and in 2012-2014 (with HAM-Nat) are compared. On the basis of the results of exploratory interviews, university entrants in winter semester 2013/2014 participated in a written survey on why they chose their subject and place of study and their expectations of their course of study.

**Results: **No problems were encountered in introducing the extended selection procedure that included the HAM-Nat Test. The HAM-Nat had a great influence on the selection decision. About 65% of the students admitted would not have obtained a place if the decision had been based exclusively on their Abitur grade [grade obtained in the German school-leaving examination]. On average, male applicants obtained better HAM-Nat results than female ones.

The questionnaire was answered by 147 out of 191 university entrants (77%). In the case of applicants from Saxony-Anhalt, the principle reasons for choosing the regional capital are its proximity, the social environment offered, good conditions for studying and the feel-good factor at the university. For the majority of applicants, however, particularly applicants from other federal states, the relatively good chances of admission in Magdeburg were the main reason.

**Conclusion: **The Magdeburg Medical Faculty regards the HAM-Nat as a suitable tool for selecting applicants with outstanding knowledge of natural sciences and thus of increasing and harmonising levels of knowledge at the start of the course. Completion of the standard period of study and success in the 1st part of the German Medical Examination will be the subject of further observation of the students. The HAM-Nat, as a performance-related selection procedure, is not suitable for giving active preference to natives of Saxony-Anhalt in the application procedure but their number has increased since it was introduced. Applicants primarily use the selection procedure tactically to obtain the university place they want to study medicine. Specifics relating to curricula and university profile and research areas are not critical to their choice.

## Zusammenfassung

**Hintergrund/Zielsetzung:** Die Medizinische Fakultät Magdeburg hat zum Wintersemester 2012/13 einen Wissenstest zur Auswahl ihrer Studienbewerber eingeführt. Das Hamburger Auswahlverfahren für medizinische Studiengänge - Naturwissenschaftsteil (HAM-Nat) umfasst einen Multiple Choice-Test mit Fragen zu medizinisch relevanten Aspekten der Fächer Biologie, Physik, Chemie und Mathematik, der speziell für die Auswahl von Medizinbewerbern entwickelt wurde.

Es wird untersucht, wie der HAM-Nat die Studierendenauswahl beeinflusst, aus welchen Gründen sich Studierende im Rahmen des Bewerbungsverfahrens für die Teilnahme am Test entscheiden und welche Erwartungen sie an das Studium haben.

**Methodik: **Die Auswahlverfahren der Hochschule 2011 (ohne HAM-Nat) und 2012-2014 (mit HAM-Nat) werden verglichen. Aufbauend auf den Ergebnissen explorativer Interviews wurden erstmalig Studienanfänger des Wintersemesters 2013/2014 zur Wahl von Studienfach und Studienort sowie zu ihren Erwartungen an das Studium schriftlich befragt.

**Ergebnisse: **Das um den HAM-Nat-Test erweiterte Auswahlverfahren wurde problemlos eingeführt. Der HAM-Nat hatte einen großen Einfluss auf die Auswahlentscheidung. Rund 65% der Zugelassenen hätten bei ausschließlicher Berücksichtigung der Abiturnote keinen Studienplatz erhalten. Männliche Bewerber zeigten im Durchschnitt bessere HAM-Nat-Ergebnisse als weibliche.

Den Fragebogen beantworteten 147 von 191 Studienanfängern(77%). Für Studienbewerber aus Sachsen-Anhalt sind Hauptgründe für die Wahl der Landeshauptstadt die regionale Nähe, das vorhandene soziale Umfeld, die guten Studienbedingungen und der Wohlfühlcharakter am Studienort. Für die Mehrzahl der Bewerber, insbesondere für Bewerber aus anderen Bundesländern, standen dagegen die relativ guten Zulassungschancen in Magdeburg im Vordergrund.

**Schlussfolgerung:** Die Medizinische Fakultät Magdeburg sieht im HAM-Nat ein geeignetes Instrument, um Bewerber mit hervorragenden naturwissenschaftlichen Kenntnissen auszuwählen und so den Kenntnisstand zu Beginn des Studiums zu heben und zu harmonisieren. Die Einhaltung der Regelstudienzeit und der Erfolg im 1. Abschnitt der Ärztlichen Prüfung werden Gegenstand weiterer Beobachtung der Studierenden sein. Als leistungsbezogener Auswahltest ist der HAM-Nat nicht geeignet, Landeskinder im Zulassungsverfahren aktiv zu bevorzugen, dennoch hat ihre Anzahl nach Einführung des HAM-Nat zugenommen. Die Bewerber nutzen das Auswahlverfahren vorrangig taktisch, um ihren Wunschstudienplatz Medizin zu erhalten. Curriculare Spezifika bzw. die Profil- und Forschungsschwerpunkte der Universität sind nicht auswahlentscheidend.

## Background

German faculties of medicine can award 60 percent of the university places available after pre-allocated places have been deducted through a university selection procedure (AdH) with the addition of the places as yet unfilled from the quota reserved for the best *Abitur* results. The selection procedure should be primarily performance-based [http://www.landesrecht.sachsen-anhalt.de/jportal/?quelle=jlink&query=HSchulZulG+ST&psml=bssahprod.psml&max=true&aiz=true]. As well as the *Abitur* grade obtained (“level of qualification”), the individual grades given on the *Abitur* certificate and/or

1. the results of a subject-specific study aptitude test,
2. the nature of professional training or occupation and/or
3. the result of a selection interview can be taken into account in selection.

The potential selection tools differ by the extent to which the *Abitur* grade continues to dominate as a performance criterion and in the logistics they require. None of the criteria has been validated prospectively in relation to professional success [1]. 

### The selection procedure in Magdeburg

Up to the 2011/12 winter semester, the *Abitur* grade obtained was the sole selection criterion applied in the selection procedure used at the Magdeburg Medical Faculty. Because *Abitur* grades obtained in the individual German federal states are not directly comparable however [2], the faculty decided to introduce a test of knowledge from winter semester 2012/13 onwards for the selection of applicants^1^. The natural sciences test used at the Medical Faculty Hamburg (HAM-Nat) is a multiple-choice test with questions on the aspects of biology, physics, chemistry and mathematics that are relevant to medicine, which was specifically developed and tested for the selection of medicine applicants [3], [4], [5], [6], [7]. The Hamburg Medical Faculty introduced the selection test primarily to reduce drop-outs from the 1st part of the course [3]. The HAM-Nat gives candidates with outstanding knowledge of natural sciences higher chances of admission. The aim is to increase and harmonise performance level in the first semesters, particularly in the area of natural sciences. It is also intended to increase the number of students from the largest student group (the 60% selected by the university selection procedure) who take the 1st part of the German Medical Examination at the proper time. 

According to the regulations governing university capacity, 191 to 193 students annually begin to study medicine in Magdeburg, approx. 150 of whom are selected by the faculty itself through the university selection procedure taking waiting lists into account. Until 2011, selection was based on *Abitur* grade alone. In the new selection procedure, from 2012 onwards 700 applicants who put Magdeburg as their first choice, are identified in a pre-selection procedure and ranked by the average *Abitur* grade obtained in their university entrance qualification. Applicants in places 1-25 are admitted directly to the course i.e. without taking the test (quota of outstanding students). From place 26 onwards, university places are awarded on the basis of the average *Abitur* grade combined with the result of the selection test. For this purpose, the average *Abitur* grade is calculated as a linear score ranging between 60 (for an *Abitur* grade of 1.0) and 0 (from a grade 4.0 onwards). Up to 59 points are allocated to the result of the HAM-Nat test. From place 26 onwards, an applicant is ranked by the total of the two scores [http://www.med.uni-magdeburg.de/Studierende/Studieng%C3%A4nge/Humanmedizin/Bewerbung+und+Zulassung/Hochschulauswahlverfahren+Humanmedizin+%28hochschulstart_de%29-p-13682.html]. When students have the same overall score - as also occurred in earlier years - voluntary service (e.g. voluntary military service, federal voluntary service) recognised under University Admissions Foundation regulations [http://www.hochschulstart.de/fileadmin/downloads/Gesetze/g03.pdf] is taken into account. When students are ranked equally, a random selection procedure is applied. 

In a first stage, this study compares the university’s selection procedure from 2011 to 2014 in relation to number of applicants, the *Abitur* grade obtained by applicants and those admitted, their gender distribution, place of origin, test results and the change in rankings made on the basis of the test. Finally, the students selected are characterised in relation to their application behaviour and their further study plans. As soon as results of the state examination M1 are available for further cohorts, a subsequent study will investigate how the aims of the faculty

1. harmonising entrance requirements (easier teaching),
2. reducing the quota of drop-outs and
3. promoting completion of the standard period of study are achieved with the introduction of the new selection procedure.

## Methods

### Selection procedure 

The selection procedures applied at the university in 2011 (without HAM-Nat) and in 2012-2014 (with HAM-Nat) are compared in relation to participant statistics and test results. The internal consistency [8] of the selection test (Cronbach’s α) was determined to assess reliability. 

The HAM-Nat test is an economical procedure in terms of personnel and time and is therefore cost-effective. In Magdeburg, approx. 45 members of staff are simultaneously involved in administering the test in ten lecture rooms. Preparation for the test consists of setting the questions, the question review process and co-ordinating the questions at the three sites involved (Hamburg, Magdeburg, Berlin). The tests are a paper-based assessment analysed by a standardised procedure (IMS, KLAUS). The test statistic is available on the day of the test, which means that decisions can be taken about items which may be of questionable quality. Test participants receive the decision on their admission very quickly. All results and the assessment are replicable so that the test procedure provides legal certainty.

#### Student Survey

In the initial stage of a two-stage qualitative-quantitative methodological procedure, 17 female and 15 male university entrants who had taken part in the 2012 or 2013 university selection procedure were interviewed. The interview was analysed using the basic techniques of qualitative content analysis [9], [10].

On the basis of the results of these interviews and in line with the results of the student survey [11], [12], the university entrants of the 2013/14 winter semester were asked to complete a written questionnaire anonymously as part of the introductory event. The questionnaire contained 36 questions predominantly based on a 5-point Likert scale “1= does not apply at all” to 5= “fully applies” with free text fields. It is given in full in the appendix with response frequencies.

Overall 147 out of 191 university entrants (77%) from all admission quotas completed the standardised questionnaire. A response with scores 4 and 5 is considered below to express agreement with a statement. The data collected were analysed using SPSS Statistics version 21. 

## Results

### Comparison of the selection procedures applied from 2011 to 2014

To demonstrate the influence of the HAM-Nat procedure on the composition of the students admitted, the selection procedures used in 2012-2014 were compared with those used in 2011. Up to and including 2011, applicants expressing all preferences for location were admitted in the order of their *Abitur* grades up to the cut-off ranking. In 2011, the cut-off grade was 1.5. In the HAM-Nat procedure, the 700 applicants with the best *Abitur* grades who had made Magdeburg their 1st preference were pre-selected. Admissions were awarded on the basis of *Abitur* grade and HAM-Nat result up to the cut-off ranking. Because not all the students admitted enrol in the first year but intake capacity has to be completely used up, the number of those admitted by the university selection procedure is greater than 60% of the fixed intake capacity. 

From 2011 to 2014, the number of medicine applicants who had put Magdeburg as their 1st preference remained the same after a decrease related to the introduction of the HAM-Nat (see Table 1 (Tab. 1)). Because the number of applicants with *Abitur* grades in the 1.3-1.7 range, whose chances of admission are significantly changed by a good test result, has substantially increased, from 2013 onwards there was an improvement in the *Abitur* grade up to which applicants were invited to sit the HAM-Nat in Magdeburg (in 2012 students with *Abitur* grades to 2.9 were invited while from 2013 onwards students with *Abitur* grades to 2.0 were invited). The *Abitur* grade which would have been good enough for admission had selection been continued on the basis of *Abitur* grade only remained about the same from 2012 to 2014. 

In 2012, only 58% of the applicants invited to sit the HAM-Nat actually sat the test; since 2013 the quota of participants has been approx. 80%. The average *Abitur* grade obtained by those admitted has become lower i.e. applicants with less good *Abitur* grades have been given a chance on the basis of their good test results (see Table 1 (Tab. 1)). Applicants with very good *Abitur* grades but little knowledge of natural science have lost their chance of admission. This is in line with the intention of the procedure to select applicants with outstanding knowledge in the natural sciences. In 2012 and 2013, the HAM-Nat result shows weak correlation with the *Abitur* grade (i.e. applicants with low *Abitur* grades have high test scores), but this is not the case in 2014, although the internal consistency of the test is almost the same in all three test years (Cronbach’s α 0.87 to 0.89).

**Figure 1:**
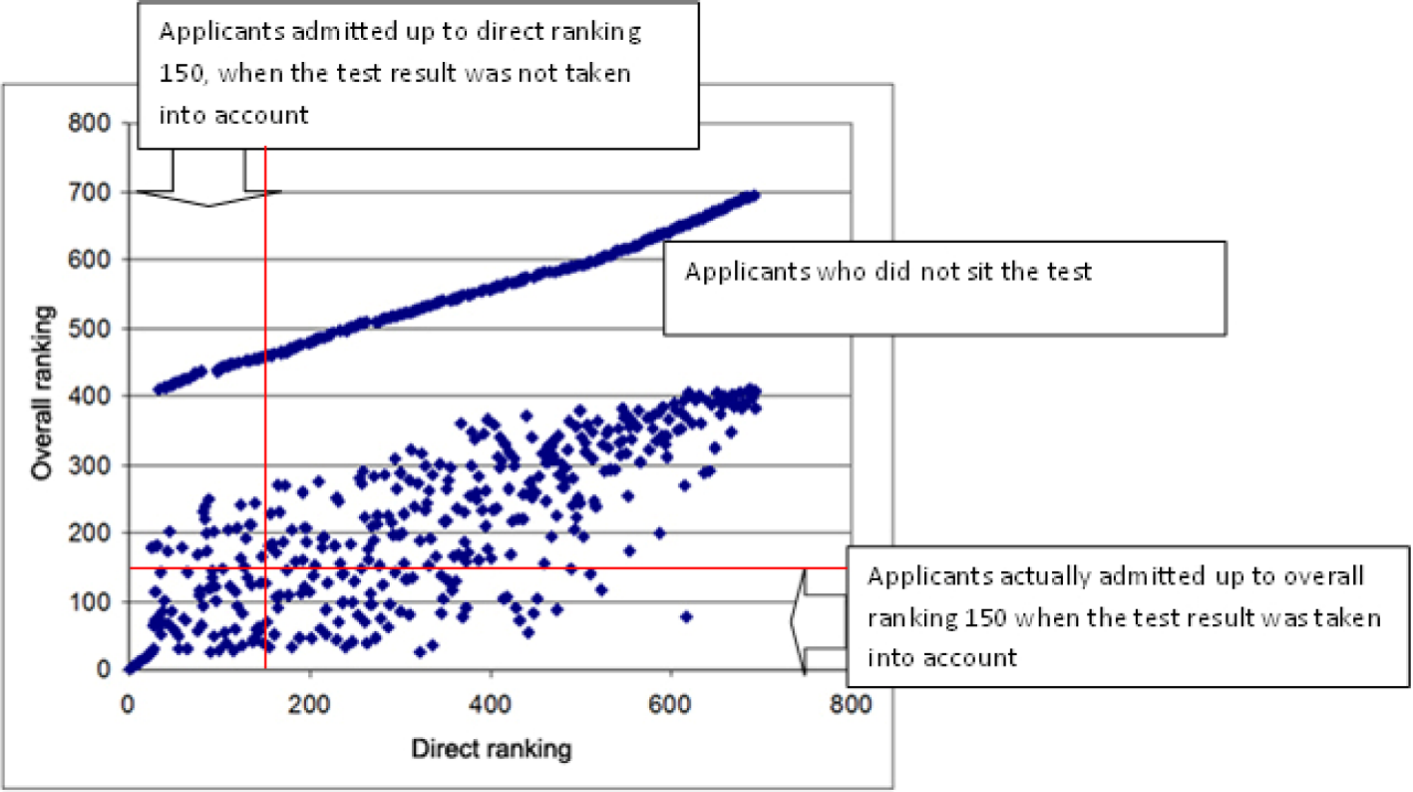
Rankings of 694 female applicants before (direct ranking; on the basis of Abitur grade only) and after (overall ranking based on *Abitur* grade and test result) the selection test, 2012 procedure

**Figure 2:**
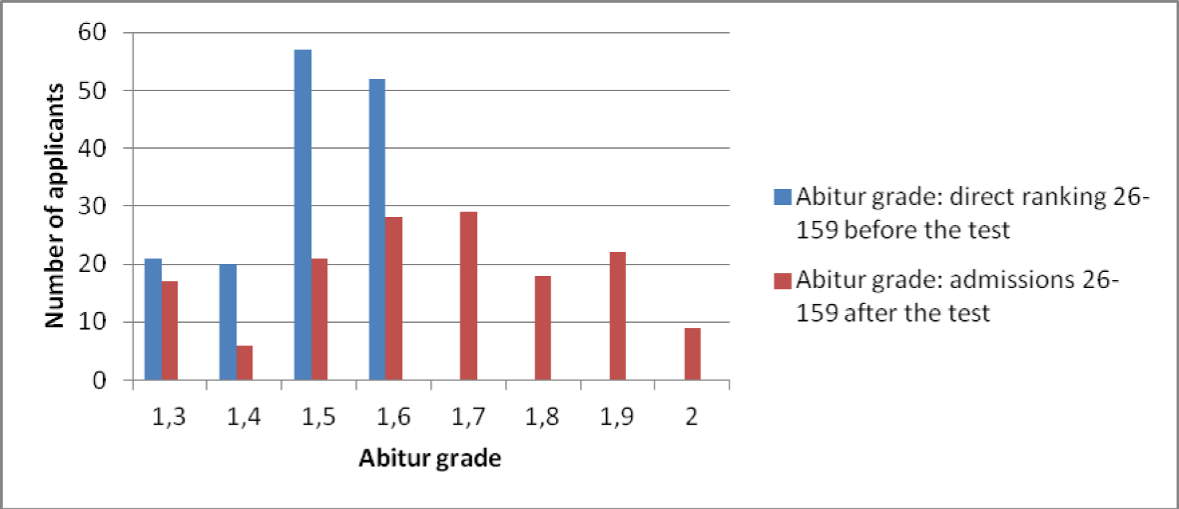
Distribution of *Abitur* grades obtained by applicants with rankings 26 -159 before (direct ranking) and after (overall ranking) the selection test, 2014 procedure

The selection efficacy of the tests, measured by the Paternoster effect [13], increased, particularly between the first and second year of the test. The “Paternoster effect” quantifies the proportion of those admitted who were offered a place only when the HAM-Nat result was taken into account, out of all those admitted. In 2012 it was 56% (see Table 1 (Tab. 1) and Figure 1 (Fig. 1)) and subsequently increased to about two thirds (see Figure 2 (Fig. 2)). The increase can be explained by the improvement in cut-off *Abitur* grade at which applicants were invited to take the HAM-Nat test, which meant that the differences in the *Abitur* grades of the test participants had less effect on selection. 

Figure 1 (Fig. 1) shows the Paternoster effect. Test participants with a direct ranking up to 150 (on the left of the vertical line) would have obtained a place before the introduction of the HAM-Nat. After taking the test result into account, participants with an overall ranking up to 150 (under the horizontal line), but a direct ranking above 150 (i.e. applicants in the lower right quadrant), are the “winners” from the new selection procedure, because they are admitted only when their good test results are taken into consideration. Participants in the lower left quadrant have retained in the test their pre-existing chance of admission based on *Abitur* grade. Despite the fact that their *Abitur* grade of 1.2-1.5 made them eligible for admission, applicants in the upper left quadrant were refused a place because of their test results. Applicants who did not sit the test were given, as statutorily required, overall rankings after test participants.

Male participants achieved about 4 points more than female ones in the HAM-Nat in all three years. This meant that the proportion of male students admitted increased compared with a procedure based on *Abitur* grade only.

#### 2013 Written Student Survey 

##### Choosing to study medicine

For some of the university entrants achieving the best possible *Abitur* grade (54% agreement) or being well prepared for a future university course (38%) was already important when they chose their advanced courses at school, but the most important factor was generally interest in the content of the advanced course (93%).

One third of those interviewed felt that their school had prepared them well for the HAM-Nat. This value was above average in the case of students from science or technology specialist schools (52%) and of those who had recently passed the *Abitur* (43%). 86% of university entrants who have passed the selection test see preparing for the test as helpful for further studies as well.

**Figure 3:**
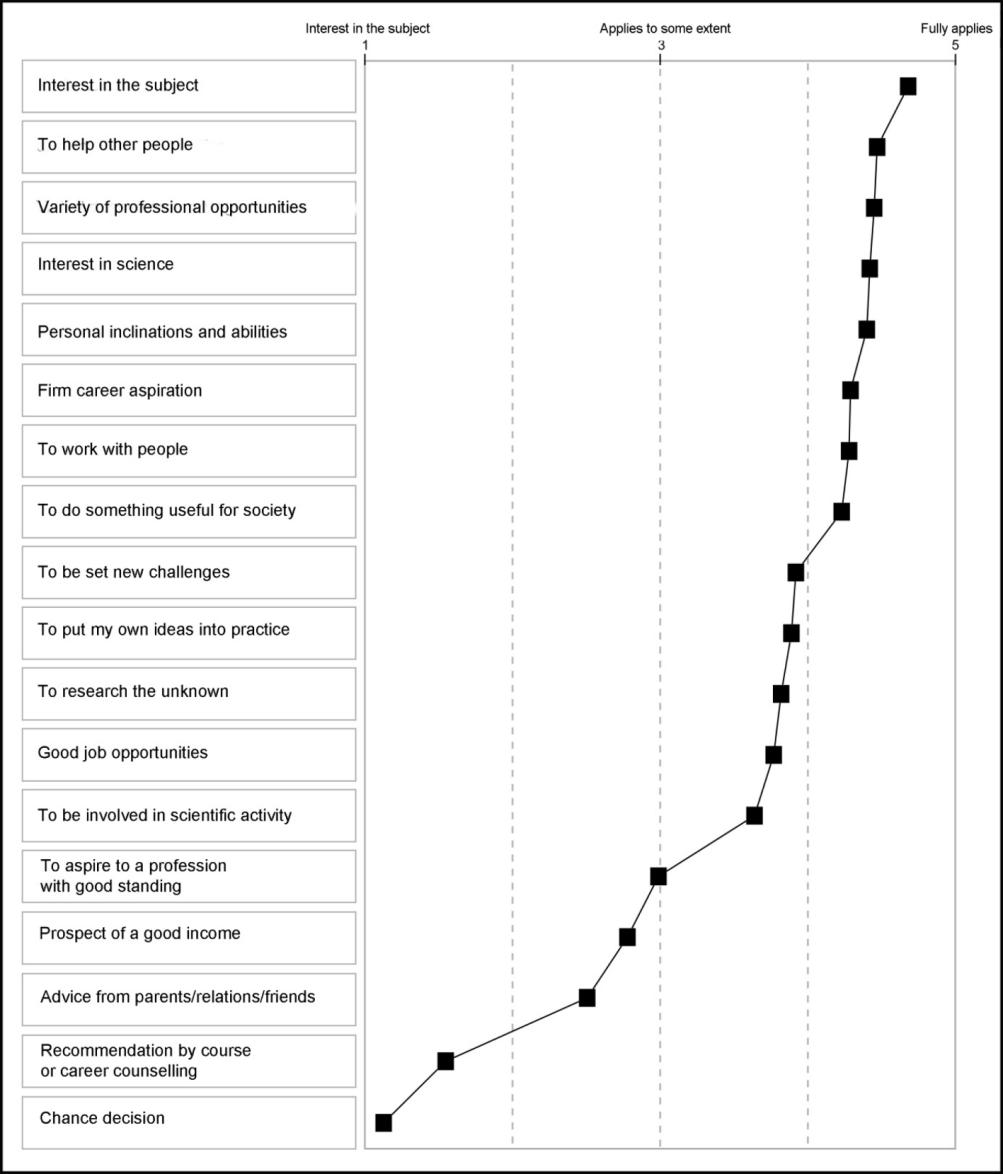
Reasons for choosing to study medicine, N=140, mean values for response format 1 “doesn’t apply at all” to 5 “fully applies”. Points are connected to make the profile clearer

Important reasons for taking up the study of medicine are, besides a dominant specific interest in the subject, the opportunity to help people, to work with people and to do something useful for the community that being a doctor provides (see Figure 3 (Fig. 3)). Although interest in the natural sciences was highly rated, later scientific activity plays a subordinate role only. 90 per cent of those interviewed are attracted by the variety of professional opportunities; personal inclinations and abilities are almost as important. 7 out of 10 future doctors were certain they wanted to enter this profession even before they had finished school. 

In addition, analysis of the interviews demonstrated that, besides the variety of opportunities on the job market, the prospect of a high income was obviously critical for the decision to study medicine, although this was described as important by only 17 percent of university entrants completing the written questionnaire, an indication of socially desirable response behaviour in the written test. 

46 out of 127 university entrants (36%) stated that they had at least one parent who worked in the health services. For 18 of them (39%) advice from parents, relations or friends was important in choosing a subject, while only 17% of the university entrants whose parents were not in the health services mentioned this. The father’s advice appeared to make more of an impression than the mother’s.

##### Expectations of the course

At the start of the course, all students are optimistic about succeeding (98% agreement). High practical relevance (96%), the acquisition of much factual knowledge and “hard work” (86%) are associated with the course. Although scientific activity plays only a subordinate role in the choice of subject, the university entrants expect scientific work (79%), discussion of theoretical questions (78%) or orientation towards research (69%) to form part of their studies. Analysis of the 11^th^ Student Survey [12] showed that the students of human medicine wanted primarily to work with people. For effectively every interviewee the most important aim was to succeed in their studies and then to find an area of work that they enjoyed (97%), to acquire lasting knowledge (95%) and to live up to their own standards (94%). 85 per cent of future doctors also consider a life outside their studies to be important.

##### Reasons for the choice of university

**Figure 4:**
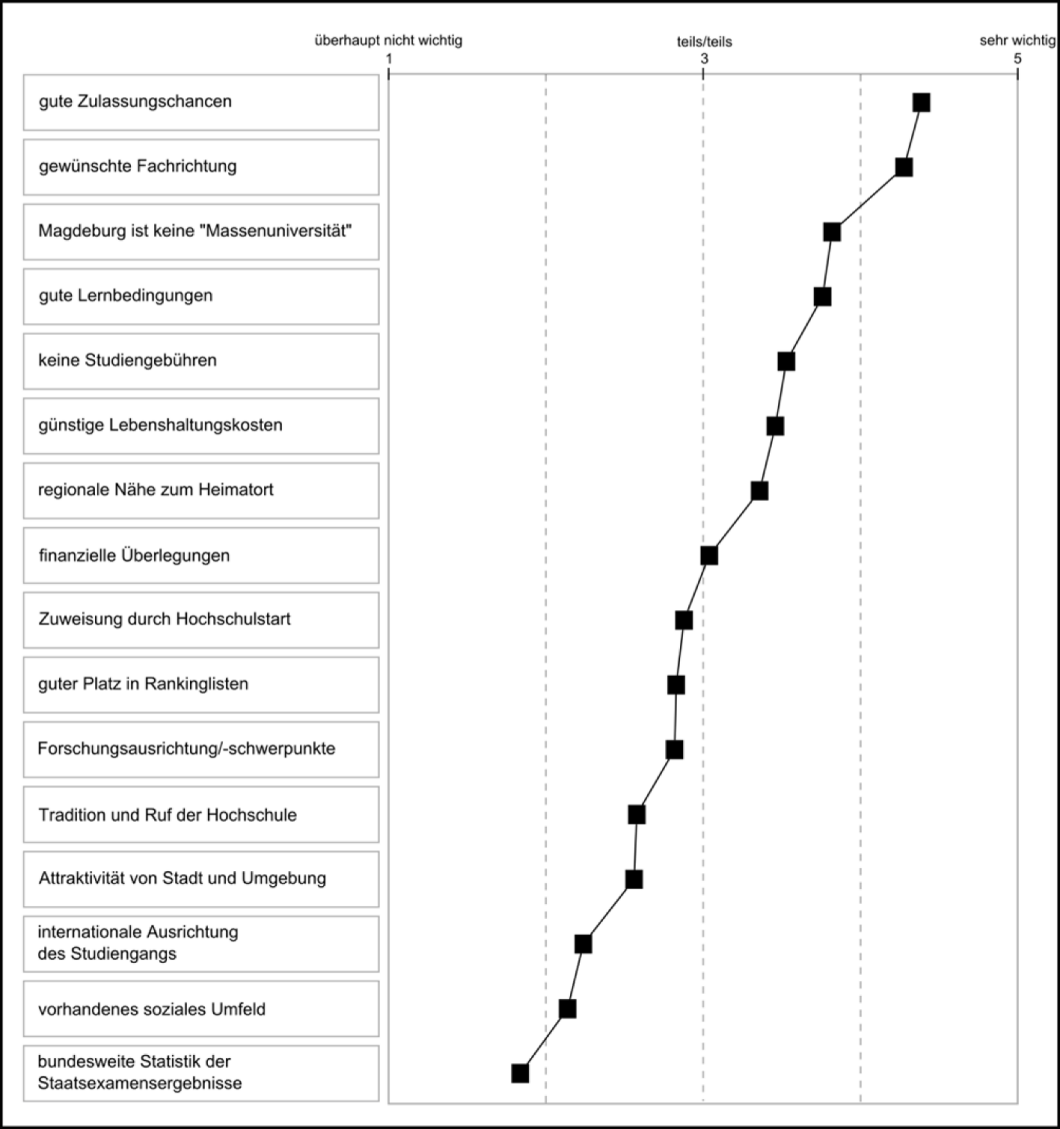
Reasons for choosing the Otto-von-Guericke University as the place to study, N=135, mean values for response format 1 “doesn’t apply at all” to 5 “fully applies”. Points are connected to make the profile clearer

The main reason for applying to the Medical Faculty in Magdeburg is the relatively good chance of being admitted to study medicine (see Figure 4 (Fig. 4)). Hence Magdeburg is considered a good choice because of the lower student numbers, good learning conditions and lack of tuition fees. For almost one applicant in two proximity to the home town is important.

Most university entrants said that they come from Saxony-Anhalt (N=42), North Rhine-Westphalia (N=20), Berlin (N=17), Bavaria (N=15) or Lower Saxony (N=12). While the university entrants from Saxony-Anhalt appreciate regional proximity and the social environment offered, the high chances of admission were more important for the remaining interviewees. 

It had already become evident in the preliminary interviews that Magdeburg was often chosen for tactical reasons. The city was considered by the interviewees less as a place to live than as a place to study which provides the necessary conditions for graduating in medicine. 

## Discussion

No problems were associated with introducing the HAM-Nat test as selection procedure at the Medical Faculty Magdeburg. Acceptance by participants is high: 86% of university entrants regard the HAM-Nat as helping them in their further studies. After the number of applicants naming Magdeburg as their first preference had decreased substantially and all applicants with an *Abitur* grade up to 2.9 had been invited to sit the test in 2012, the year the HAM-Nat was introduced, the number of applicants almost doubled in the subsequent year thus returning to the level of applications received in 2011. Evidently it took a year for applicant behaviour to adjust to the new selection procedure. In 2013 in comparison with 2012, despite the better average *Abitur* grade obtained by the HAM-Nat participants (the cut-off grade for an invitation to sit the test was now an *Abitur* grade of 2.0) the students admitted via the university selection procedure had a worse average *Abitur* grade (going from a mean grade of 1.55 to 1.68). The high Paternoster effect at two thirds clearly shows that a good test result leads to admission and that applicants with weaker *Abitur* grades also have a chance of admission. This was intended. 

Male applicants obtain substantially better HAM-Nat results on average than female applicants, which means that more male applicants are now admitted than was the case earlier (see Table 1 (Tab. 1)). This phenomenon also occurs in Hamburg as well as with other aptitude tests e.g. in Graz [14], [15]. One reason could be that male applicants show greater interest in natural sciences which is already evident in the advanced courses chosen at school. Our 2013 Student Survey shows that male students are more likely than female students to have chosen mathematics (male: 45%/female: 31%), physics (10%/4%) or chemistry (15%/10%) as an advanced course at school. The latter tend to choose German (15%/39%), English (27%/33%) and biology (49%/50%). With this subject profile female school-leavers obtain better grades in the *Abitur* than males (e.g. in 2011 in North Rhine-Westphalia about 60% more women than men were awarded an *Abitur* grade of 1.0 to 1.9 [http://www.it.nrw.de/statistik/analysen/stat_studien/2012/band_75/wl_z089201254.html). As a result, the proportion of women in the applicant population is greater than that of men [16]. The emphasis of the HAM-Nat test is different from that of an admission procedure based on *Abitur* grades alone.

In the case of applicants from Saxony-Anhalt, the principle reasons for their choice of university, besides regional proximity and the social environment offered, are the good conditions for studying and the feel-good factor at the university. The faculty’s public relations in the region focussed on a positive presentation of the university; it should be maintained to ensure new new physicians for Saxony-Anhalt particularly for rural areas. For applicants from other federal states with an average *Abitur* grade >1.6 in particular, the relatively good chances of admission, if they have good prior knowledge of the natural sciences, are the most important factor. Thus preferences are used tactically by approx. 80% of medicine applicants to obtain the university place they want. Specifics relating to curricula and key profile and research areas are not critical for their application.

With the introduction of the HAM-Nat the number of Saxony-Anhalt applicants admitted to Magdeburg initially doubled (see Table 1 (Tab. 1)). It reduced again in subsequent years, which was predominantly caused by the increased proportion of applicants from other federal states. The HAM-Nat, as a performance-related selection procedure, is not suitable for giving preference to natives of Saxony-Anhalt in the application procedure if they do not perform better than other applicants. The law governing university medical education and the regulations of the University Admissions Foundation have not given and still do not give preferential treatment to local applicants in the university selection procedure http://www.landesrecht-hamburg.de/jportal/portal/page/bshaprod.psml?showdoccase=1&doc.id=jlr-VergabeVStiftVHArahmen&st=lr], [http://wcms.uzi.uni-halle.de/download.php?down=28395&elem=2554892] in accordance with the prohibition of discrimination in article 3 par. 3 of the Basic Law. If students admitted from outside Saxony-Anhalt return to their home states after university, medical care within the region may be put at risk more than previously by the lack of young doctors. Only 64 out of 138 university entrants surveyed (46%) can envisage setting up a local practice after university. Of these 23% are considering becoming a general practitioner. Only 9% would like to practice as a general practitioner in a rural region, which is the reason for concern about future healthcare in rural areas [see also [17]]. 

No conclusion can be drawn on the basis of this study on the effect of the HAM-Nat selection test with regard to the success achieved in their studies by those selected. In the future, further analyses will study the parameters of successful study, particularly the number of drop-outs and the results of the 1st part of the German Medical Examination (M1). The M1 State Examination tests basic knowledge in the natural sciences which is also the target of the HAM-Nat test. Since it is impossible to compare the results of the test with those of the 1st part of the Medical Examination obtained by our HAM-Nat partners in Hamburg and Berlin because of the pilot courses there, it is all the more interesting to continue to observe how successful the Magdeburg HAM-Nat cohorts are in their studies.

## Appendix

Questionnaires (see attachment 1 ) and response frequencies (see attachment 2 ) giving mean values. 

## Notes

^1^ In the text below, the masculine pronoun only is used for the sake of simplicity. The feminine pronoun is obviously always included.

## Acknowledgements

The authors are greatly indebted to the colleagues they consulted for their advice, particularly to

- The members of the selection panel,
- The Dean of the Medical Faculty, Prof. Dr. H.-J. Rothkötter,
- The Dean for Student Affairs in the Medical Faculty, Prof. Dr. C. H. Lohmann
- Prof. Dr. G. Reiser,
- The interviewer S. Hartwig and
- The students who took part

for their opinions and ideas . We thank the Medical Faculty for its financial support for the teaching project.

## Competing interests

The authors declare that they have no competing interests.

## Supplementary Material

Questionnaire in German (Fragebogen zur "Qualitätsverbesserung des Verfahrens zur Auswahl von Studienplatzbewerbern der Medizinischen Fakultät" (QUAMED))

Results (Evaluationsergebnisse Erstsemesterbefragung 2013)

## Figures and Tables

**Table 1:**
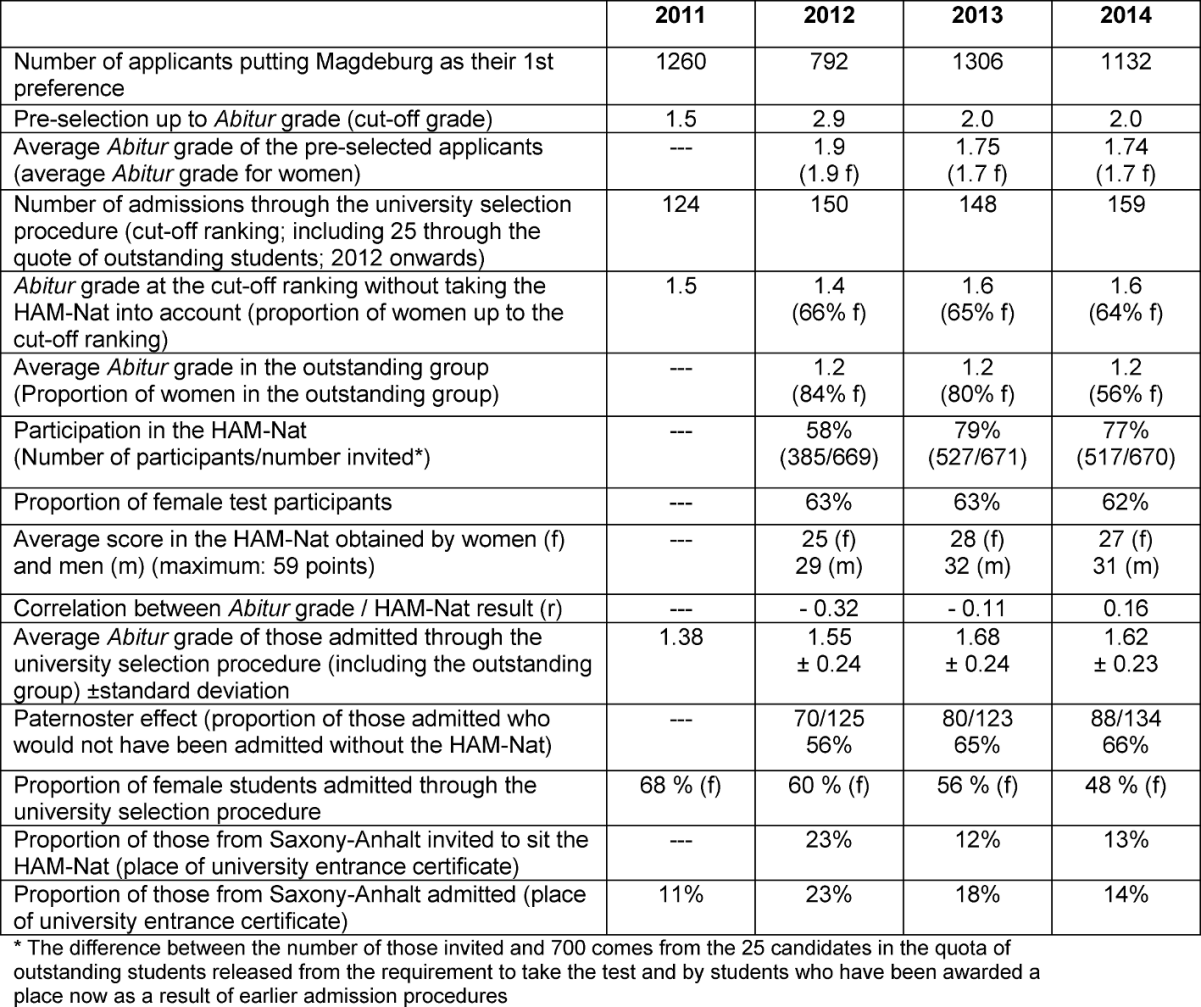
Comparison of the selection procedures in 2012-2014 (with HAM-Nat) with 2011 (selection by *Abitur* grade exclusively)
