# Supplementary material for: Introduction of the HAM-Nat examination – applicants and students admitted to the Medical Faculty in 2012-2014
Source: GMS Z Med Ausbild. 2015 Nov 16;32(5):Doc53. doi: 10.3205/zma000995 (PMC4647160; doi:10.3205/zma000995)
Supplement: Questionnaire in German (Fragebogen zur "Qualitätsverbesserung des Verfahrens zur Auswahl von Studienplatzbewerbern der Medizinischen Fakultät" (QUAMED)) [file ZMA-32-53-s-001.pdf]

Fragebogen zur „Qualitätsverbesserung des Verfahrens zur Auswahl von Studienplatzbewerbern der Medizinischen Fakultät“ (QUAMED)

Liebe Studentinnen und Studenten,

herzlich willkommen an der Medizinischen Fakultät der Otto-von-Guericke-Universität Magdeburg!

Um Sie auch in Zukunft angemessen beraten und auch das Auswahlverfahren optimieren zu können, möchten wir Sie bitten, den Fragebogen auszufüllen. Uns interessieren Ihre Kriterien, die Sie bewogen haben, an unserer Fakultät Humanmedizin zu studieren.

Nehmen Sie sich bitte die Zeit, den Fragebogen zu beantworten. Wählen Sie die Antwortvorgaben aus, die Ihren persönlichen Erfahrungen und Ihren eigenen Ansichten am nächsten kommen.

Ihre Angaben werden völlig anonym behandelt. Selbstverständlich ist Ihre Teilnahme an der Erhebung freiwillig. Die Studierenden, die bereits Ihre Einwilligung an der Teilnahme an dem Forschungsvorhaben zur Qualitätsverbesserung des Verfahrens zur Auswahl von Studienplatzbewerbern im Rahmen des Auswahlverfahrens der Hochschule (HAM-Nat) gegeben haben, werden am Ende des Fragebogen gebeten, Ihre Matrikel-Nummer anzugeben.

Prof. Dr. med. C. H. Lohmann
Studiendekan der Medizinischen Fakultät

1. In welchem Jahr haben Sie die Hochschulreife erworben?

☐ 2013 ☐ 2012 ☐ 2011 ☐ 2010 ☐ 2009 ☐ 2008 ☐ 2007 ☐ 2006 ☐ 2005 ☐ 2004

☐ anderes Jahr: [ ]

2. In welchem Bundesland haben Sie die Hochschulreife erworben? Oder war das im Ausland?

☐ Baden-Württemberg ☐ Bayern ☐ Berlin
☐ Brandenburg ☐ Bremen ☐ Hamburg
☐ Hessen ☐ Mecklenburg-Vorpommern ☐ Niedersachsen
☐ Nordrhein-Westfalen ☐ Rheinland-Pfalz ☐ Saarland
☐ Sachsen ☐ Sachsen-Anhalt ☐ Schleswig-Holstein
☐ Thüringen ☐ Ausland ☐ Sonstiges

3. Mit welcher Durchschnittsnote haben Sie die Hochschulreife erworben?

Tragen Sie in die obere Zeile die Einerstelle ein und in die untere die Zehntelstelle ein.

Beispiel 1: Wenn Ihre Durchschnittsnote z.B. 1,0 ist, kreuzen Sie oben x1 und unten x0 an.
Beispiel 2: Wenn Ihre Durchschnittsnote z.B. 2,3 ist, kreuzen Sie oben x2 und unten x3 an.

	x0	x1	x2	x3	x4	x5	x6	x7	x8	x9
1er	☐	☐	☐	☐	☐	☐	☐	☐	☐	☐
0.1er	☐	☐	☐	☐	☐	☐	☐	☐	☐	☐

4. Welches Profil verfolgte die Ausbildungsstätte, in der Sie die Hochschulzugangsberechtigung erworben haben?

☐ naturwissenschaftlich/ technisch ☐ sprachlich
☐ sportlich/ musisch/ künstlerisch ☐ alternativ pädagogisch (Waldorf, Montessori)
☐ evangelisch/ katholisch/ ökumenisch ☐ kein spezielles Profil

5. In welchen Kursen haben Sie Ihre Hochschulreife abgelegt?

1. Leistungskurs: [|]
2. Leistungskurs: [|]
1. Grundkurs: [|]
2. Grundkurs: [|]

01 Deutsch/Literatur
02 Englisch
03 Französisch
04 andere neue Sprachen
05 Latein
06 Griechisch
07 Kunst/Musik
08 anderes sprachlich-literarisch-künstlerisches Fach
09 Mathematik
10 Informatik
11 Physik
12 Chemie
13 Biologie
14 Technologie/Technik
15 anderes mathematisch-naturwissenschaftliches Fach
16 Erdkunde/Geographie
17 Geschichte/Gemeinschaftskunde
18 Wirtschafts-/Sozialwissenschaften
19 Erziehungswissenschaft, Philosophie künstlerisches Fach
20 anderes gesellschafts-/sozialwissenschaftliches Fach
21 Sport
22 Religion

6. Nach welchem Kriterium haben Sie Ihre Leistungskurse ausgewählt?

	trifft überhaupt nicht zu	trifft wenig zu	teils/ teils	trifft ziemlich zu	trifft völlig zu	keine Angabe
Erreichung der bestmöglichen Abiturnote	☐	☐	☐	☐	☐	☐
Interesse am Inhalt des Faches	☐	☐	☐	☐	☐	☐
gute Vorbereitung auf das zukünftige Studium	☐	☐	☐	☐	☐	☐
Empfehlung durch Angehörige/Bekannte	☐	☐	☐	☐	☐	☐
fachliche Unterstützung durch Angehörige/Bekannte	☐	☐	☐	☐	☐	☐
vorgegebenes Angebot der Ausbildungsstätte	☐	☐	☐	☐	☐	☐

7. Bitte nennen Sie uns Ihre <u>zuletzt</u> ausgeübte Tätigkeit direkt vor der Aufnahme des Studiums.

☐ Erwerb der Hochschulreife
☐ Zivil-, Wehrdienst, Soziales Jahr, Ökologisches Jahr
☐ anderes Studium
☐ Auslandsaufenthalt
☐ Berufsausbildung/ -tätigkeit
☐ Sonstiges

8. Besitzen Sie Vorerfahrungen im medizinischen Bereich (Mehrfachnennungen möglich)?

☐ Nein
☐ Ja, im Rahmen eines Freiwilligendienstes
☐ Ja, im Rahmen von Berufserfahrung
☐ Ja, im Rahmen von Praktika
☐ Ja, im Rahmen einer Ausbildung

9. Haben Sie sich im Vorfeld an einer anderen Universität beworben?

☐ Ja
☐ Nein

10. Was waren Ihre ersten drei Ortspräferenzen auf dem Zulassungsantrag der Quote, über die Sie zugelassen wurden?

1.
2.
3.

11. Über welche Quote wurden Sie zum Medizinstudium zugelassen?

Abiturbestenquote ☐ --> weiter mit Frage 14
AdH-Auswahlverfahren der Hochschule (HAM-Nat und Exzellenzquote) ☐ --> weiter mit Frage 12
Wartezeitquote ☐ --> weiter mit Frage 14
Sonstiges ☐ --> weiter mit Frage 14

12. Wie fühlten Sie sich durch die Schule auf den HAM-Nat-Test vorbereitet?

☐ mangelhaft ☐ ausreichend ☐ befriedigend ☐ gut ☐ sehr gut

13. Denken Sie, dass die Vorbereitungen auf den Auswahltest Ihnen auch im zukünftigen Medizinstudium von Nutzen sein werden?

☐ ja ☐ nein

14. Ist das Medizinstudium Ihr ursprüngliches "Wunschfach"?

☐ Medizin ist mein Wunschfach ☐ Ich hatte keinen speziellen Studienwunsch

☐ Ich hätte lieber folgendes Fach studiert: ____________

15. Wie zutreffend sind folgende Gründe für Ihre Wahl eines Medizinstudium?

	trifft überhaupt nicht zu	trifft wenig zu	teils/ teils	trifft ziemlich zu	trifft völlig zu	keine Angabe
persönliche Neigungen und Begabungen	☐	☐	☐	☐	☐	☐
naturwissenschaftliches Interesse	☐	☐	☐	☐	☐	☐
Aussicht auf ein hohes Einkommen	☐	☐	☐	☐	☐	☐
Vielfalt der beruflichen Möglichkeiten	☐	☐	☐	☐	☐	☐
Ratschläge von Eltern/Verwandten/Freunden	☐	☐	☐	☐	☐	☐
Fachinteresse	☐	☐	☐	☐	☐	☐
Streben nach einem angesehenen Beruf	☐	☐	☐	☐	☐	☐
Empfehlung von Studien- oder Berufsberatung	☐	☐	☐	☐	☐	☐
fester Berufswunsch	☐	☐	☐	☐	☐	☐
gute Arbeitsmarktchancen	☐	☐	☐	☐	☐	☐
zufällige Entscheidung	☐	☐	☐	☐	☐	☐
mit Menschen arbeiten	☐	☐	☐	☐	☐	☐
anderen Menschen helfen	☐	☐	☐	☐	☐	☐
Nützliches für die Allgemeinheit tun	☐	☐	☐	☐	☐	☐
Unbekanntes erforschen	☐	☐	☐	☐	☐	☐
Wissenschaftlich tätig sein	☐	☐	☐	☐	☐	☐
neue Aufgaben gestellt bekommen	☐	☐	☐	☐	☐	☐
eigene Ideen verwirklichen	☐	☐	☐	☐	☐	☐

16. Sie haben sich für ein Studium an der Otto-von-Guericke Universität entschieden. Wie wichtig waren Ihnen folgende Gründe für diese Wahl?

	überhaupt nicht wichtig	kaum wichtig	teils/ teils	ziemlich wichtig	sehr wichtig	keine Angabe
Tradition und Ruf der Hochschule	☐	☐	☐	☐	☐	☐
vorhandenes soziales Umfeld (Partner, Freunde,...)	☐	☐	☐	☐	☐	☐
Attraktivität von Stadt und Umgebung	☐	☐	☐	☐	☐	☐
gute Zulassungschancen	☐	☐	☐	☐	☐	☐
regionale Nähe zum Heimatort	☐	☐	☐	☐	☐	☐
günstige Lebenshaltungskosten	☐	☐	☐	☐	☐	☐

16. Sie haben sich für ein Studium an der Otto-von-Guericke Universität entschieden. Wie wichtig waren Ihnen folgende Gründe für diese Wahl? (Fortsetzung)

	überhaupt nicht wichtig	kaum wichtig	teils/ teils	ziemlich wichtig	sehr wichtig	keine Angabe
gewünschte Fachrichtung	☐	☐	☐	☐	☐	☐
Forschungsausrichtung/-schwerpunkte	☐	☐	☐	☐	☐	☐
finanzielle Überlegungen	☐	☐	☐	☐	☐	☐
guter Platz in Rankinglisten	☐	☐	☐	☐	☐	☐
bundesweite Statistik der Staatsexamensergebnisse	☐	☐	☐	☐	☐	☐
internationale Ausrichtung des Studienganges	☐	☐	☐	☐	☐	☐
gute Lernbedingungen	☐	☐	☐	☐	☐	☐
keine Studiengebühren	☐	☐	☐	☐	☐	☐
Zuweisung durch Hochschulstart	☐	☐	☐	☐	☐	☐
Magdeburg ist keine "Massenuniversität"	☐	☐	☐	☐	☐	☐

17. Welche Informationsquellen haben Sie vor der Entscheidung für Magdeburg herangezogen?

		herangezogen	
Auskünfte im Freundeskreis	☐ nicht	☐ teilweise	☐ intensiv
Hinweise innerhalb der Familie	☐ nicht	☐ teilweise	☐ intensiv
Homepage der Universität	☐ nicht	☐ teilweise	☐ intensiv
individuelle Studienberatung	☐ nicht	☐ teilweise	☐ intensiv
Internetrecherche (z.B. in Foren)	☐ nicht	☐ teilweise	☐ intensiv
Agentur für Arbeit	☐ nicht	☐ teilweise	☐ intensiv
Tag der offenen Tür/ Campus Days	☐ nicht	☐ teilweise	☐ intensiv
Schnupperstudium	☐ nicht	☐ teilweise	☐ intensiv
Lange Nacht der Wissenschaft	☐ nicht	☐ teilweise	☐ intensiv

Sonstiges: ______________________

18. Welche Erwartungen haben Sie an Ihr Studium/ an das Studienfach?

	trifft überhaupt nicht zu	trifft wenig zu	teils/ teils	trifft ziemlich zu	trifft völlig zu	keine Angabe
großes Faktenwissen erwerben	☐	☐	☐	☐	☐	☐
Prüfungen bestehen	☐	☐	☐	☐	☐	☐
viel und intensiv arbeiten	☐	☐	☐	☐	☐	☐
sich mit theoretischen Fragen auseinandersetzen	☐	☐	☐	☐	☐	☐
Praxisbezug	☐	☐	☐	☐	☐	☐
wissenschaftlich arbeiten	☐	☐	☐	☐	☐	☐
Forschungsbezug	☐	☐	☐	☐	☐	☐

19. Wie wichtig ist Ihnen?

	überhaupt nicht wichtig	kaum wichtig	teils/ teils	ziemlich wichtig	sehr wichtig	keine Angabe
die Vereinbarkeit von Studium und Familie	☐	☐	☐	☐	☐	☐
die Vereinbarkeit von Studium und Nebenjob/Beruf	☐	☐	☐	☐	☐	☐
Möglichkeit eines Teilzeitstudiums	☐	☐	☐	☐	☐	☐

20. Wie beabsichtigen Sie Ihr Studium zu finanzieren?

Ersparnisse/Eigenkapital	☐ nicht	☐ teilweise	☐ hauptsächlich
Unterstützung durch die Eltern/Verwandte	☐ nicht	☐ teilweise	☐ hauptsächlich
Unterstützung durch den/die Partner/in	☐ nicht	☐ teilweise	☐ hauptsächlich
BAföG	☐ nicht	☐ teilweise	☐ hauptsächlich
eigener Verdienst/ Job	☐ nicht	☐ teilweise	☐ hauptsächlich
Stipendium	☐ nicht	☐ teilweise	☐ hauptsächlich
Studentenkredit	☐ nicht	☐ teilweise	☐ hauptsächlich
Kindergeld	☐ nicht	☐ teilweise	☐ hauptsächlich
soziale Unterstützung (z.B. Sozialhilfe, Wohngeld)	☐ nicht	☐ teilweise	☐ hauptsächlich

21. Beabsichtigen Sie, während des Studiums zu arbeiten?

☐ Nein

☐ Ja, in der vorlesungsfreien Zeit	-->	☐ ≤20	☐ ≤40	☐ ≤60	☐ ≤80	☐ >80	Stunden im Monat
☐ Ja, in der Vorlesungszeit	-->	☐ ≤20	☐ ≤40	☐ ≤60	☐ ≤80	☐ >80	Stunden im Monat
☐ Ja, während des gesamten Semesters	-->	☐ ≤20	☐ ≤40	☐ ≤60	☐ ≤80	☐ >80	Stunden im Monat

☐ Weiß ich noch nicht

22. Wie informiert fühlen Sie sich über folgende Bereiche?

	gar nicht	wenig	ausreichend	gut	sehr gut	interessiert mich nicht
Studien- und Prüfungsordnung	☐	☐	☐	☐	☐	☐
Möglichkeiten der Studienberatung	☐	☐	☐	☐	☐	☐
Einzelheiten des BAföG	☐	☐	☐	☐	☐	☐
Möglichkeiten für ein Studium im Ausland	☐	☐	☐	☐	☐	☐
Arbeitsmarktsituation im angestrebten Tätigkeitsfeld	☐	☐	☐	☐	☐	☐
aktuelle politische Überlegungen der Hochschulentwicklung	☐	☐	☐	☐	☐	☐

23. Wie schätzen Sie die zukünftig an Sie gestellten Studienanforderungen ein?

fachliches Niveau	☐ zu gering	☐ genau richtig	☐ zu hoch	☐ kann ich nicht beurteilen
Umfang des Stoffes	☐ zu gering	☐ genau richtig	☐ zu hoch	☐ kann ich nicht beurteilen
Zeitintensität (Gesamtaufwand Studium, Lehrveranstaltungen und Lernzeit)	☐ zu gering	☐ genau richtig	☐ zu hoch	☐ kann ich nicht beurteilen

24. Wie wichtig sind Ihnen folgende Aspekte im Studium?

	überhaupt nicht wichtig	kaum wichtig	teils/ teils	ziemlich wichtig	sehr wichtig	keine Angabe
Abschluss des Studiums in der Regelstudienzeit	☐	☐	☐	☐	☐	☐
Bestehen des Studiums	☐	☐	☐	☐	☐	☐
den eigenen Maßstäben gerecht werden	☐	☐	☐	☐	☐	☐
einen Bereich finden der Spaß macht	☐	☐	☐	☐	☐	☐
Promotionsthema bearbeiten mit dem Ziel, einen Doktortitel zu erlangen	☐	☐	☐	☐	☐	☐
ein breites Allgemeinwissen aneignen	☐	☐	☐	☐	☐	☐
nachhaltiges Wissen erlangen	☐	☐	☐	☐	☐	☐
ein "Leben" neben dem Studium	☐	☐	☐	☐	☐	☐

25. Aus Ihrer heutigen Sicht, in welcher Fachdisziplin möchten Sie tätig sein (Einfachnennung)?

☐ Chirurgie ☐ Innere Medizin ☐ Allgemeinmedizin ☐ Kinderheilkunde

☐ Frauenheilkunde ☐ andere Fachrichtung, und zwar: [ ]

☐ noch nicht darüber nachgedacht

26. Wenn Sie sich jetzt für Ihre Zukunft entscheiden müssten, welche Richtung würden Sie wählen (Einfachnennung)?

eigene Niederlassung	☐	--> weiter mit Frage 25
Angestellte/r in einer Klinik	☐	--> weiter mit Frage 27
Angestellter in einer Praxis/Medizinisches Versorgungszentrum	☐	--> weiter mit Frage 27
Arbeit in der Wissenschaft/Forschung	☐	--> weiter mit Frage 27
Angestellte/r im öffentlichen Gesundheitsdienst Gesundheitsdienst (MDK, Krankenkasse, Gesundheitsamt,...)	☐	--> weiter mit Frage 27

27. Wenn Sie sich für eine Niederlassung entscheiden würden. Welcher Bereich würde Ihnen mehr zusagen?

☐ Niederlassung als Hausarzt ☐ Niederlassung als Spezialist

28. Wo würden Sie sich am ehesten niederlassen?

☐ In einer Großstadt (>100.000 Einwohner) ☐ in einer mittelgroßen Stadt (20.000-100.000 Einwohner)

☐ in einer Kleinstadt (<20.000 Einwohner) ☐ in einer ländlichen Region

29. Jetzt würden wir gerne von Ihnen wissen, wo Sie sich selbst in 10 Jahren sehen.

30. Nennen Sie uns bitte Ihr Geschlecht.

☐ weiblich ☐ männlich

31. In welchem Jahr wurden Sie geboren?

☐ 1996 ☐ 1995 ☐ 1994 ☐ 1993 ☐ 1992 ☐ 1991 ☐ 1990 ☐ 1989 ☐ 1988 ☐ 1987

☐ anderes Geburtsjahr:

32. Welche Staatsangehörigkeit besitzen Sie?

☐ Deutsche Staatsangehörigkeit ☐ sonstige EU-Staatsangehörigkeit

☐ Doppelte Staatsangehörigkeit ☐ Nicht-EU-Staatsangehörigkeit

33. Bitte geben Sie Ihren Familienstand an.

☐ ledig ☐ verheiratet/ in Partnerschaft lebend ☐ geschieden/ verwitwet

34. Wie viele Kinder haben Sie?

☐ keine ☐ 1 Kind ☐ 2 Kinder ☐ mehr als 2 Kinder

35. Nennen Sie uns bitte den höchsten berufsqualifizierenden Abschluss Ihrer Eltern.

Vater:

- ☐ keinen beruflichen Abschluss
- ☐ Lehre bzw. Facharbeiterabschluss
- ☐ Meisterprüfung
- ☐ Fachschule, Technikerschule, Handelsakademie o.Ä.
- ☐ Fachhochschule, Spezialhochschule
- ☐ Universität, Technische Hochschule o.Ä
- ☐ sonstiges, weiß nicht

Mutter:

- ☐ keinen beruflichen Abschluss
- ☐ Lehre bzw. Facharbeiterabschluss
- ☐ Meisterprüfung
- ☐ Fachschule, Technikerschule, Handelsakademie o.Ä.
- ☐ Fachhochschule, Spezialhochschule
- ☐ Universität, Technische Hochschule o.Ä
- ☐ sonstiges, weiß nicht

36. Sind Ihre Eltern im Gesundheitswesen tätig?

Vater:

- ☐ nein
- ☐ Ärztliche Tätigkeit
- ☐ Krankenpflege
- ☐ Rettungsdienst

Mutter:

- ☐ nein
- ☐ Ärztliche Tätigkeit
- ☐ Krankenpflege
- ☐ Rettungsdienst

Teilnehmer, die an der Soziodemographischen Studie im Rahmen des hochschuleigenen Auswahlverfahrens (HAM-Nat)[2] teilgenommen haben, bitten wir an dieser Stelle Ihre Matrikel-Nummer einzutragen:

Vielen Dank für Ihre Teilnahme!

[2] Alle im Rahmen der Studie erhobenen Daten werden pseudonymisiert verarbeitet. Dies bedeutet, dass Ihr Name durch einen Code ersetzt wird. Zugang zu der Schlüsselliste mit Code und Namen hat jeweils nur ein(e) Mitarbeiter(in) des Studiendekanates sowie sein bzw. ihre Vertreter(in), zu den pseudonymisierten Daten nur der Studienleiter sowie Mitarbeiter(innen) des Studiendekanates. Die Schlüsselliste wird 6 Jahre nach Beendigung Ihres Studiums gelöscht, so dass ein Personenbezug dann nicht mehr möglich ist.
