# Supplementary material for: Introduction of the HAM-Nat examination – applicants and students admitted to the Medical Faculty in 2012-2014
Source: GMS Z Med Ausbild. 2015 Nov 16;32(5):Doc53. doi: 10.3205/zma000995 (PMC4647160; doi:10.3205/zma000995)
Supplement: Results (Evaluationsergebnisse Erstsemesterbefragung 2013) [file ZMA-32-53-s-002.pdf]

DER KANZLER

DEZERNAT
STUDIENANGELEGENHEITEN
K32

Daniel Grupski

Otto-von-Guericke-Universität Magdeburg
Universitätsplatz 2
39106 Magdeburg

Telefon: +49 391 67-11206
Telefax: +49 391 67-11140

daniel.grupski@ovgu.de
www.ovgu.de

Otto-von-Guericke-Universität Magdeburg, Postfach 4120, 39016 Magdeburg

An:
Erstsemesterbefragung
persönlich/vertraulich

Evaluationsergebnisse 2013

Sehr geehrter Herr/Sehr geehrte Frau Erstsemesterbefragung,

Sie erhalten hier die Evaluationsergebnisse Ihrer Lehrveranstaltung: 2013.
Bei Rückfragen stehe ich Ihnen gerne zur Verfügung.

Fragebogen Typ FME80.

Mit freundlichen Grüßen
Daniel Grupski

Anlage
Auswertungsbericht

Erstsemesterbefragung

2013 ()
Erfasste Fragebögen = 147

Auswertungsteil der geschlossenen Fragen

Legende

Fragetext

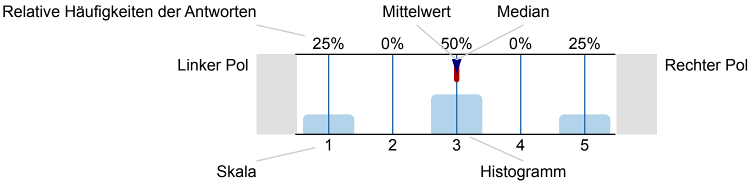

n=Anzahl
mw=Mittelwert
md=Median
E.=Enthaltung

1. In welchem Jahr haben Sie die Hochschulreife erworben?

n=145

2013	36.6%
2012	21.4%
2011	14.5%
2010	6.2%
2009	2.1%
2008	0.7%
2007	6.9%
2006	7.6%
2005	0.7%
2004	2.1%
anderes Jahr:	1.4%

2. In welchem Bundesland haben Sie die Hochschulreife erworben? Oder war das im Ausland?

n=147

Baden-Württemberg	6.8%
Bayern	10.2%
Berlin	11.6%
Brandenburg	6.1%
Hamburg	2%
Hessen	2.7%
Niedersachsen	8.2%
Nordrhein-Westfalen	13.6%
Rheinland-Pfalz	0.7%
Saarland	0.7%
Sachsen	3.4%
Sachsen-Anhalt	28.6%
Schleswig-Holstein	0.7%
Thüringen	0.7%
Ausland	4.1%

3. Mit welcher Durchschnittsnote haben Sie die Hochschulreife erworben?

n=144

0 - 19	86.1%
20 - 39	13.9%

4. Welches Profil verfolgte die Ausbildungsstätte, in der Sie die Hochschulzugangsberechtigung erworben haben?

n=131

naturwissenschaftlich/ technisch	25.2%
sportlich/ musisch/ künstlerisch	6.9%
evangelisch/ katholisch/ ökumenisch	4.6%
sprachlich	14.5%
alternativ pädagogisch (Waldorf, Montessori)	0%
kein spezielles Profil	48.9%

6. Nach welchem Kriterium haben Sie Ihre Leistungskurse ausgewählt?

Skala: 1 = trifft überhaupt nicht zu … 5 = trifft völlig zu

	1	2	3	4	5	
Erreichung der bestmöglichen Abiturnote	6,6%	14,7%	25%	31,6%	22,1%	n=136 mw=3,5 md=4
Interesse am Inhalt des Faches	0,7%	2,2%	4,4%	26,5%	66,2%	n=136 mw=4,6 md=5
gute Vorbereitung auf das zukünftige Studium	9,2%	22%	30,5%	20,6%	17,7%	n=141 mw=3,2 md=3
Empfehlung durch Angehörige/Bekannte	46,3%	25,7%	19,1%	7,4%	1,5%	n=136 mw=1,9 md=2 E.=1
fachliche Unterstützung durch Angehörige/Bekannte	52,6%	20,4%	19%	5,8%	2,2%	n=137 mw=1,8 md=1 E.=2
vorgegebenes Angebot der Ausbildungsstätte	45,1%	12,8%	22,6%	12%	7,5%	n=133 mw=2,2 md=2 E.=4

7. Bitte nennen Sie uns Ihre zuletzt ausgeübte Tätigkeit direkt vor der Aufnahme des Studiums.

n=137

Erwerb der Hochschulreife	33.6%
anderes Studium	24.8%
Berufsausbildung/ -tätigkeit	24.8%
Zivil-, Wehrdienst, Soziales Jahr,	8.8%
Auslandsaufenthalt	2.9%
Sonstiges	5.1%

8. Besitzen Sie Vorerfahrungen im medizinischen Bereich (Mehrfachnennungen möglich)?

n=147

Nein	19.7%
Ja, im Rahmen von Praktika	63.9%
Ja, im Rahmen eines Freiwilligendienstes	12.9%
Ja, im Rahmen einer Ausbildung	21.1%
Ja, im Rahmen von Berufserfahrung	15%

9. Haben Sie sich im Vorfeld an einer anderen Universität beworben?

n=146

Ja	48.6%
Nein	51.4%

11. Über welche Quote wurden Sie zum Medizinstudium zugelassen?

n=144

Abiturbestenquote	5.6%
AdH-Auswahlverfahren der Hochschule (HAM-Nat und Exzellenzquote)	68.1%
Wartezeitquote	16%
Sonstiges	10.4%

12. Wie fühlten Sie sich durch die Schule auf den HAM-Nat-Test vorbereitet?

n=92

mangelhaft	12%
ausreichend	15.2%
befriedigend	41.3%
gut	18.5%
sehr gut	13%

13. Denken Sie, dass die Vorbereitungen auf den Auswahltest Ihnen auch im zukünftigen Medizinstudium von Nutzen sein werden?

n=94

ja	86.2%
nein	13.8%

14. Ist das Medizinstudium Ihr ursprüngliches Wunschfach?

n=146

Medizin ist mein Wunschfach	97.3%
Ich hatte keinen speziellen Studienwunsch	2.1%
Ich hätte lieber folgendes Fach studiert:	0.7%

15. Wie zutreffend sind folgende Gründe für Ihre Wahl eines Medizinstudium?

Item	Linker Pol	1	2	3	4	5	Rechter Pol	Statistik
persönliche Neigungen und Begabungen	trifft überhaupt nicht zu	0%	0,7%	5,7%	50%	43,6%	trifft völlig zu	n=140 mw=4,4 md=4
naturwissenschaftliches Interesse	trifft überhaupt nicht zu	0%	0,7%	14,5%	33,8%	51%	trifft völlig zu	n=145 mw=4,4 md=5
Aussicht auf ein hohes Einkommen	trifft überhaupt nicht zu	7%	27,5%	47,9%	14,8%	2,8%	trifft völlig zu	n=142 mw=2,8 md=3 E.=1
Vielfalt der beruflichen Möglichkeiten	trifft überhaupt nicht zu	0,7%	2,1%	7,9%	38,6%	50,7%	trifft völlig zu	n=140 mw=4,4 md=5
Ratschläge von Eltern/Verwandten/Freunden	trifft überhaupt nicht zu	23,7%	26,6%	24,5%	14,4%	10,8%	trifft völlig zu	n=139 mw=2,6 md=2 E.=1
Fachinteresse	trifft überhaupt nicht zu	0,7%	0,7%	1,4%	28%	69,2%	trifft völlig zu	n=143 mw=4,6 md=5
Streben nach einem angesehenen Beruf	trifft überhaupt nicht zu	9,9%	27%	27%	27,7%	8,5%	trifft völlig zu	n=141 mw=3 md=3
Empfehlung von Studien- oder Berufsberatung	trifft überhaupt nicht zu	57%	20,7%	9,6%	8,1%	4,4%	trifft völlig zu	n=135 mw=1,8 md=1 E.=4
fester Berufswunsch	trifft überhaupt nicht zu	2,8%	7,1%	15,6%	27,7%	46,8%	trifft völlig zu	n=141 mw=4,1 md=4 E.=2
gute Arbeitsmarktchancen	trifft überhaupt nicht zu	0,7%	7,6%	26,4%	47,9%	17,4%	trifft völlig zu	n=144 mw=3,7 md=4 E.=1
zufällige Entscheidung	trifft überhaupt nicht zu	87,9%	8,6%	2,9%	0,7%	0%	trifft völlig zu	n=140 mw=1,2 md=1
mit Menschen arbeiten	trifft überhaupt nicht zu	0,7%	3,5%	11,9%	44,1%	39,9%	trifft völlig zu	n=143 mw=4,2 md=4
anderen Menschen helfen	trifft überhaupt nicht zu	0,7%	1,4%	6,3%	40,1%	51,4%	trifft völlig zu	n=142 mw=4,4 md=5

Nützliches für die Allgemeinheit tun

trifft überhaupt nicht zu	1	2	3	4	5	trifft völlig zu	
	1,4%	4,2%	15,5%	39,4%	39,4%		n=142 mw=4,1 md=4

Unbekanntes erforschen

trifft überhaupt nicht zu	1	2	3	4	5	trifft völlig zu	
	2,1%	9,2%	28,4%	31,2%	29,1%		n=141 mw=3,8 md=4

Wissenschaftlich tätig sein

trifft überhaupt nicht zu	1	2	3	4	5	trifft völlig zu	
	2,1%	9,2%	32,6%	36,9%	19,1%		n=141 mw=3,6 md=4

neue Aufgaben gestellt bekommen

trifft überhaupt nicht zu	1	2	3	4	5	trifft völlig zu	
	2,1%	1,4%	25,7%	47,2%	23,6%		n=144 mw=3,9 md=4

eigene Ideen verwirklichen

trifft überhaupt nicht zu	1	2	3	4	5	trifft völlig zu	
	2,8%	4,2%	23,8%	46,2%	23,1%		n=143 mw=3,8 md=4

16. Sie haben sich für ein Studium an der Otto-von-Guericke Universität entschieden. Wie wichtig waren Ihnen folgende Gründe für diese Wahl?

Tradition und Ruf der Hochschule

überhaupt nicht wichtig	1	2	3	4	5	sehr wichtig	
	19,8%	28,2%	26,7%	17,6%	7,6%		n=131 mw=2,6 md=3 E.=10

vorhandenes soziales Umfeld (Partner, Freunde,...)

überhaupt nicht wichtig	1	2	3	4	5	sehr wichtig	
	43,6%	9,8%	11,3%	11,3%	24,1%		n=133 mw=2,6 md=2 E.=10

Attraktivität von Stadt und Umgebung

überhaupt nicht wichtig	1	2	3	4	5	sehr wichtig	
	23,5%	22%	33,3%	15,9%	5,3%		n=132 mw=2,6 md=3 E.=7

gute Zulassungschancen

überhaupt nicht wichtig	1	2	3	4	5	sehr wichtig	
	5,1%	8,8%	12,4%	19%	54,7%		n=137 mw=4,1 md=5 E.=8

regionale Nähe zum Heimatort

überhaupt nicht wichtig	1	2	3	4	5	sehr wichtig	
	30,4%	5,9%	15,6%	17%	31,1%		n=135 mw=3,1 md=3 E.=7

günstige Lebenshaltungskosten

überhaupt nicht wichtig	1	2	3	4	5	sehr wichtig	
	13%	10,9%	25,4%	33,3%	17,4%		n=138 mw=3,3 md=4 E.=5

gewünschte Fachrichtung

überhaupt nicht wichtig	1	2	3	4	5	sehr wichtig	
	6,2%	9,3%	15,5%	20,9%	48,1%		n=129 mw=4 md=4 E.=6

Forschungsausrichtung/-schwerpunkte

überhaupt nicht wichtig	1	2	3	4	5	sehr wichtig	
	15,4%	25%	28,7%	25%	5,9%		n=136 mw=2,8 md=3 E.=7

finanzielle Überlegungen

überhaupt nicht wichtig	1	2	3	4	5	sehr wichtig	
	13%	18,1%	35,5%	22,5%	10,9%		n=138 mw=3 md=3 E.=4

guter Platz in Rankinglisten

überhaupt nicht wichtig	1	2	3	4	5	sehr wichtig	
	19,5%	19,5%	30,1%	22,6%	8,3%		n=133 mw=2,8 md=3 E.=7

bundesweite Statistik der Staatsexamensergebnisse

überhaupt nicht wichtig	1	2	3	4	5	sehr wichtig	
	42%	27,5%	13,7%	13,7%	3,1%		n=131 mw=2,1 md=2 E.=11

internationale Ausrichtung des Studienganges

überhaupt nicht wichtig	1	2	3	4	5	sehr wichtig	
	31,5%	24,6%	26,9%	13,1%	3,8%		n=130 mw=2,3 md=2 E.=9

gute Lernbedingungen

überhaupt nicht wichtig	1	2	3	4	5	sehr wichtig	
	9%	7,5%	16,5%	49,6%	17,3%		n=133 mw=3,6 md=4 E.=6

keine Studiengebühren

überhaupt nicht wichtig	1	2	3	4	5	sehr wichtig	
	18,1%	8,7%	19,6%	31,2%	22,5%		n=138 mw=3,3 md=4 E.=4

Zuweisung durch Hochschulstart

überhaupt nicht wichtig	1	2	3	4	5	sehr wichtig	
	32,1%	10,1%	19,3%	15,6%	22,9%		n=109 mw=2,9 md=3 E.=25

Magdeburg ist keine Massenuniversität

überhaupt nicht wichtig	1	2	3	4	5	sehr wichtig	
	7,4%	5,9%	23,5%	37,5%	25,7%		n=136 mw=3,7 md=4 E.=5

17. Welche Informationsquellen haben Sie vor der Entscheidung für Magdeburg herangezogen?

Auskünfte im Freundeskreis

		n=139
nicht	44.6%	
teilweise	38.8%	
intensiv	16.5%	

Hinweise innerhalb der Familie

		n=139
nicht	47.5%	
teilweise	30.2%	
intensiv	22.3%	

Homepage der Universität

		n=139
nicht	16.5%	
teilweise	54%	
intensiv	29.5%	

individuelle Studienberatung

nicht	79.9%	n=134
teilweise	14.9%	
intensiv	5.2%	

Internetrecherche (z.B. in Foren)

nicht	32.1%	n=134
teilweise	36.6%	
intensiv	31.3%	

Agentur für Arbeit

nicht	96.4%	n=138
teilweise	2.9%	
intensiv	0.7%	

Tag der offenen Tür/ Campus Days

nicht	85.4%	n=137
teilweise	7.3%	
intensiv	7.3%	

Schnupperstudium

nicht	96.3%	n=135
teilweise	0.7%	
intensiv	3%	

Lange Nacht der Wissenschaft

nicht	92%	n=137
teilweise	3.6%	
intensiv	4.4%	

18. Welche Erwartungen haben Sie an Ihr Studium/ an das Studienfach?

großes Faktenwissen erwerben

	1	2	3	4	5		
trifft überhaupt nicht zu	0,7%	0,7%	2,9%	40,6%	55,1%	trifft völlig zu	n=138<br>mw=4,5<br>md=5

Prüfungen bestehen

	1	2	3	4	5		
trifft überhaupt nicht zu	0,7%	0%	1,4%	30,3%	67,6%	trifft völlig zu	n=142<br>mw=4,6<br>md=5

viel und intensiv arbeiten

	1	2	3	4	5		
trifft überhaupt nicht zu	0,7%	0%	13,1%	46,7%	39,4%	trifft völlig zu	n=137<br>mw=4,2<br>md=4

sich mit theoretischen Fragen auseinandersetzen

	1	2	3	4	5		
trifft überhaupt nicht zu	0,7%	2,1%	19,3%	47,1%	30,7%	trifft völlig zu	n=140<br>mw=4,1<br>md=4<br>E.=1

Praxisbezug

trifft überhaupt nicht zu	1	2	3	4	5	trifft völlig zu
	0,7%	0,7%	2,9%	30,2%	65,5%	

n=139
mw=4,6
md=5

wissenschaftlich arbeiten

trifft überhaupt nicht zu	1	2	3	4	5	trifft völlig zu
	0%	1,4%	19,3%	46,4%	32,9%	

n=140
mw=4,1
md=4

Forschungsbezug

trifft überhaupt nicht zu	1	2	3	4	5	trifft völlig zu
	0%	3,6%	27%	40,9%	28,5%	

n=137
mw=3,9
md=4

19. Wie wichtig ist Ihnen?

die Vereinbarkeit von Studium und Familie

überhaupt nicht wichtig	1	2	3	4	5	sehr wichtig
	5,1%	12,5%	25%	31,6%	25,7%	

n=136
mw=3,6
md=4
E.=1

die Vereinbarkeit von Studium und Nebenjob/Beruf

überhaupt nicht wichtig	1	2	3	4	5	sehr wichtig
	18,1%	25,4%	28,3%	21,7%	6,5%	

n=138
mw=2,7
md=3
E.=1

Möglichkeit eines Teilzeitstudiums

überhaupt nicht wichtig	1	2	3	4	5	sehr wichtig
	70,1%	21,2%	7,3%	0%	1,5%	

n=137
mw=1,4
md=1
E.=6

20. Wie beabsichtigen Sie Ihr Studium zu finanzieren?

Ersparnisse/Eigenkapital

nicht	21.5%
teilweise	63.7%
hauptsächlich	14.8%

n=135

Unterstützung durch die Eltern/Verwandte

nicht	7.4%
teilweise	29.4%
hauptsächlich	63.2%

n=136

Unterstützung durch den/die Partner/in

nicht	91.8%
teilweise	6%
hauptsächlich	2.2%

n=134

BAföG

nicht	70.6%
teilweise	14.7%
hauptsächlich	14.7%

n=136

eigener Verdienst/ Job

nicht	36%	n=136
teilweise	55.1%	
hauptsächlich	8.8%	

Stipendium

nicht	73.3%	n=135
teilweise	22.2%	
hauptsächlich	4.4%	

Studentenkredit

nicht	93.2%	n=133
teilweise	4.5%	
hauptsächlich	2.3%	

Kindergeld

nicht	43%	n=135
teilweise	47.4%	
hauptsächlich	9.6%	

soziale Unterstützung (z.B. Sozialhilfe, Wohngeld)

nicht	93.3%	n=135
teilweise	5.9%	
hauptsächlich	0.7%	

21. Beabsichtigen Sie, während des Studiums zu arbeiten?

Nein	22.5%	n=129
Ja, in der vorlesungsfreien Zeit	24.8%	
Ja, in der Vorlesungszeit	2.3%	
Ja, während des gesamten Semesters	15.5%	
Weiß ich noch nicht	34.9%	

Ja, in der vorlesungsfreien Zeit

	26.8%	n=41
	29.3%	
	26.8%	
	12.2%	
>80	4.9%	

Ja, in der Vorlesungszeit	Es wird keine Auswertung angezeigt, da die Anzahl der Antworten zu gering ist.
Ja, während des gesamten Semesters	Es wird keine Auswertung angezeigt, da die Anzahl der Antworten zu gering ist.

22. Wie informiert fühlen Sie sich über folgende Bereiche?

	Skala	1	2	3	4	5	Skala	Statistik
Studien- und Prüfungsordnung	gar nicht	6,5%	36,7%	26,6%	28,8%	1,4%	sehr gut	n=139 mw=2,8 md=3 E.=1
Möglichkeiten der Studienberatung	gar nicht	2,9%	21,4%	31,4%	40,7%	3,6%	sehr gut	n=140 mw=3,2 md=3 E.=1
Einzelheiten des BAföG	gar nicht	24,6%	33,6%	23,8%	16,4%	1,6%	sehr gut	n=122 mw=2,4 md=2 E.=17
Möglichkeiten für ein Studium im Ausland	gar nicht	26,1%	42,5%	18,7%	11,9%	0,7%	sehr gut	n=134 mw=2,2 md=2 E.=6
Arbeitsmarktsituation im angestrebten Tätigkeitsfeld	gar nicht	3,6%	13,7%	26,6%	41%	15,1%	sehr gut	n=139 mw=3,5 md=4 E.=1
aktuelle politische Überlegungen der Hochschulentwicklung	gar nicht	5%	37,9%	25,7%	22,9%	8,6%	sehr gut	n=140 mw=2,9 md=3

23. Wie schätzen Sie die zukünftig an Sie gestellten Studienanforderungen ein?

fachliches Niveau

n=138

zu gering	0%
genau richtig	53.6%
zu hoch	5.8%
kann ich nicht beurteilen	40.6%

Umfang des Stoffes

n=133

zu gering	0.8%
genau richtig	41.4%
zu hoch	15%
kann ich nicht beurteilen	42.9%

Zeitintensität (Gesamtaufwand Studium,

n=135

zu gering	0%
genau richtig	29.6%
zu hoch	22.2%
kann ich nicht beurteilen	48.1%

24. Wie wichtig sind Ihnen folgende Aspekte im Studium?

Scale: 1 = überhaupt nicht wichtig … 5 = sehr wichtig

Aspekt	1	2	3	4	5	Statistik
Abschluss des Studiums in der Regelstudienzeit	1,4%	4,3%	14,5%	33,3%	46,4%	n=138, mw=4,2, md=4
Bestehen des Studiums	0%	0%	0,7%	2,9%	96,4%	n=137, mw=5, md=5
den eigenen Maßstäben gerecht werden	0%	0%	5,9%	34,6%	59,6%	n=136, mw=4,5, md=5, E.=1
einen Bereich finden der Spaß macht	0%	0%	2,9%	23,9%	73,2%	n=138, mw=4,7, md=5
Promotionsthema bearbeiten mit dem Ziel, einen Doktortitel zu erlangen	0,7%	8,1%	19,9%	40,4%	30,9%	n=136, mw=3,9, md=4, E.=2
ein breites Allgemeinwissen aneignen	0%	1,4%	14,4%	46%	38,1%	n=139, mw=4,2, md=4
nachhaltiges Wissen erlangen	0%	0,7%	4,3%	31,9%	63%	n=138, mw=4,6, md=5
ein Leben" neben dem Studium "	0%	1,4%	13,6%	34,3%	50,7%	n=140, mw=4,3, md=5

25. Aus Ihrer heutigen Sicht, in welcher Fachdisziplin möchten Sie tätig sein (Einfachnennung)?

n=122

Fachdisziplin	Anteil
Chirurgie	16.4%
Innere Medizin	16.4%
Allgemeinmedizin	8.2%
Kinderheilkunde	13.9%
Frauenheilkunde	2.5%
andere Fachrichtung, und zwar:	21.3%
noch nicht darüber nachgedacht	21.3%

26. Wenn Sie sich jetzt für Ihre Zukunft entscheiden müssten, welche Richtung würden Sie wählen (Einfachnennung)?

eigene Niederlassung	46.4%	n=138
Angestellte/r in einer Klinik	42.8%	
Angestellter in einer Praxis/Medizinisches Versorgungszentrum	5.1%	
Arbeit in der Wissenschaft/Forschung	5.8%	
Angestellte/r im öffentlichen Gesundheitsdienst	0%	

27. Wenn Sie sich für eine Niederlassung entscheiden würden. Welcher Bereich würde Ihnen mehr zusagen?

Niederlassung als Hausarzt	23.2%	n=125
Niederlassung als Spezialist	76.8%	

28. Wo würden Sie sich am ehesten niederlassen?

In einer Großstadt (>100.000 Einwohner)	35.5%	n=124
in einer mittelgroßen Stadt (20.000-100.000 Einwohner)	41.9%	
in einer Kleinstadt (	13.7%	
in einer ländlichen Region	8.9%	

30. Nennen Sie uns bitte Ihr Geschlecht.

weiblich	59.9%	n=137
männlich	40.1%	

31. In welchem Jahr wurden Sie geboren?

1996	0.7%	n=135
1995	17.8%	
1994	19.3%	
1993	18.5%	
1992	11.9%	
1991	7.4%	
1990	3%	
1989	3%	
1988	4.4%	
1987	7.4%	
anderes Geburtsjahr:	6.7%	

32. Welche Staatsangehörigkeit besitzen Sie?

Deutsche Staatsangehörigkeit	92.1%	n=139
sonstige EU-Staatsangehörigkeit	1.4%	
Nicht-EU-Staatsangehörigkeit	2.9%	
Doppelte Staatsangehörigkeit	3.6%	

33. Bitte geben Sie Ihren Familienstand an.

ledig	95.5%	n=132
verheiratet/ in Partnerschaft lebend	4.5%	
geschieden/ verwitwet	0%	

34. Wie viele Kinder haben Sie?

keine	98.5%	n=135
1 Kind	0.7%	
2 Kinder	0.7%	
mehr als 2 Kinder	0%	

35. Nennen Sie uns bitte den höchsten berufsqualifizierenden Abschluss Ihrer Eltern.

Vater:

keinen beruflichen Abschluss	1.5%	n=137
Lehre bzw. Facharbeiterabschluss	13.9%	
Meisterprüfung	5.8%	
Fachschule, Technikerschule, Handelsakademie o.Ä.	7.3%	
Fachhochschule, Spezialhochschule	8%	
Universität, Technische Hochschule o.Ä	62.8%	
sonstiges, weiß nicht	0.7%	

Mutter:

keinen beruflichen Abschluss	2.2%	n=139
Lehre bzw. Facharbeiterabschluss	31.7%	
Meisterprüfung	0%	
Fachschule, Technikerschule, Handelsakademie o.Ä.	5.8%	
Fachhochschule, Spezialhochschule	10.8%	
Universität, Technische Hochschule o.Ä	48.9%	
sonstiges, weiß nicht	0.7%	

36. Sind Ihre Eltern im Gesundheitswesen tätig?

Vater:

n=134

nein	76.9%
Ärztliche Tätigkeit	21.6%
Krankenpflege	1.5%
Rettungsdienst	0%

Mutter:

n=135

nein	74.1%
Ärztliche Tätigkeit	13.3%
Krankenpflege	11.9%
Rettungsdienst	0.7%

Profillinie

Teilbereich: Medizinische Fakultät
Name der/des Lehrenden: Erstsemesterbefragung
Titel der Lehrveranstaltung: (Name der Umfrage) 2013

Verwendete Werte in der Profillinie: Mittelwert

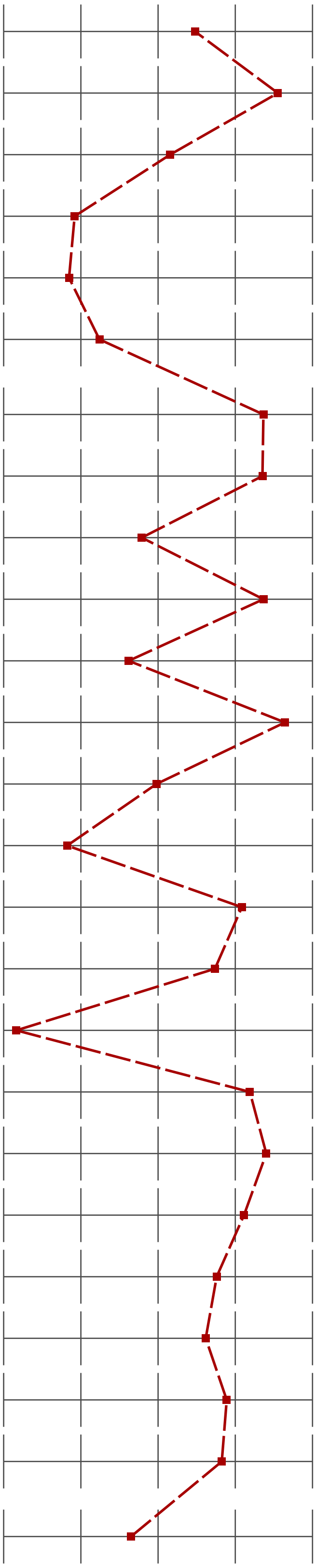

Erreichung der bestmöglichen Abiturnote	trifft überhaupt nicht zu	trifft völlig zu	n=136	mw=3,5	md=4,0	s=1,2
Interesse am Inhalt des Faches	trifft überhaupt nicht zu	trifft völlig zu	n=136	mw=4,6	md=5,0	s=0,7
gute Vorbereitung auf das zukünftige Studium	trifft überhaupt nicht zu	trifft völlig zu	n=141	mw=3,2	md=3,0	s=1,2
Empfehlung durch Angehörige/Bekannte	trifft überhaupt nicht zu	trifft völlig zu	n=136	mw=1,9	md=2,0	s=1,0
fachliche Unterstützung durch Angehörige/Bekannte	trifft überhaupt nicht zu	trifft völlig zu	n=137	mw=1,8	md=1,0	s=1,1
vorgegebenes Angebot der Ausbildungsstätte	trifft überhaupt nicht zu	trifft völlig zu	n=133	mw=2,2	md=2,0	s=1,3
persönliche Neigungen und Begabungen	trifft überhaupt nicht zu	trifft völlig zu	n=140	mw=4,4	md=4,0	s=0,6
naturwissenschaftliches Interesse	trifft überhaupt nicht zu	trifft völlig zu	n=145	mw=4,4	md=5,0	s=0,8
Aussicht auf ein hohes Einkommen	trifft überhaupt nicht zu	trifft völlig zu	n=142	mw=2,8	md=3,0	s=0,9
Vielfalt der beruflichen Möglichkeiten	trifft überhaupt nicht zu	trifft völlig zu	n=140	mw=4,4	md=5,0	s=0,8
Ratschläge von Eltern/Verwandten/Freunden	trifft überhaupt nicht zu	trifft völlig zu	n=139	mw=2,6	md=2,0	s=1,3
Fachinteresse	trifft überhaupt nicht zu	trifft völlig zu	n=143	mw=4,6	md=5,0	s=0,6
Streben nach einem angesehenen Beruf	trifft überhaupt nicht zu	trifft völlig zu	n=141	mw=3,0	md=3,0	s=1,1
Empfehlung von Studien- oder Berufsberatung	trifft überhaupt nicht zu	trifft völlig zu	n=135	mw=1,8	md=1,0	s=1,2
fester Berufswunsch	trifft überhaupt nicht zu	trifft völlig zu	n=141	mw=4,1	md=4,0	s=1,1
gute Arbeitsmarktchancen	trifft überhaupt nicht zu	trifft völlig zu	n=144	mw=3,7	md=4,0	s=0,9
zufällige Entscheidung	trifft überhaupt nicht zu	trifft völlig zu	n=140	mw=1,2	md=1,0	s=0,5
mit Menschen arbeiten	trifft überhaupt nicht zu	trifft völlig zu	n=143	mw=4,2	md=4,0	s=0,8
anderen Menschen helfen	trifft überhaupt nicht zu	trifft völlig zu	n=142	mw=4,4	md=5,0	s=0,7
Nützliches für die Allgemeinheit tun	trifft überhaupt nicht zu	trifft völlig zu	n=142	mw=4,1	md=4,0	s=0,9
Unbekanntes erforschen	trifft überhaupt nicht zu	trifft völlig zu	n=141	mw=3,8	md=4,0	s=1,0
Wissenschaftlich tätig sein	trifft überhaupt nicht zu	trifft völlig zu	n=141	mw=3,6	md=4,0	s=1,0
neue Aufgaben gestellt bekommen	trifft überhaupt nicht zu	trifft völlig zu	n=144	mw=3,9	md=4,0	s=0,9
eigene Ideen verwirklichen	trifft überhaupt nicht zu	trifft völlig zu	n=143	mw=3,8	md=4,0	s=0,9
Tradition und Ruf der Hochschule	überhaupt nicht wichtig	sehr wichtig	n=131	mw=2,6	md=3,0	s=1,2

vorhandenes soziales Umfeld (Partner, Freunde,...)	überhaupt nicht wichtig	sehr wichtig	n=133	mw=2,6	md=2,0	s=1,7
Attraktivität von Stadt und Umgebung	überhaupt nicht wichtig	sehr wichtig	n=132	mw=2,6	md=3,0	s=1,2
gute Zulassungschancen	überhaupt nicht wichtig	sehr wichtig	n=137	mw=4,1	md=5,0	s=1,2
regionale Nähe zum Heimatort	überhaupt nicht wichtig	sehr wichtig	n=135	mw=3,1	md=3,0	s=1,6
günstige Lebenshaltungskosten	überhaupt nicht wichtig	sehr wichtig	n=138	mw=3,3	md=4,0	s=1,3
gewünschte Fachrichtung	überhaupt nicht wichtig	sehr wichtig	n=129	mw=4,0	md=4,0	s=1,3
Forschungsausrichtung/-schwerpunkte	überhaupt nicht wichtig	sehr wichtig	n=136	mw=2,8	md=3,0	s=1,2
finanzielle Überlegungen	überhaupt nicht wichtig	sehr wichtig	n=138	mw=3,0	md=3,0	s=1,2
guter Platz in Rankinglisten	überhaupt nicht wichtig	sehr wichtig	n=133	mw=2,8	md=3,0	s=1,2
bundesweite Statistik der Staatsexamensergebnisse	überhaupt nicht wichtig	sehr wichtig	n=131	mw=2,1	md=2,0	s=1,2
internationale Ausrichtung des Studienganges	überhaupt nicht wichtig	sehr wichtig	n=130	mw=2,3	md=2,0	s=1,2
gute Lernbedingungen	überhaupt nicht wichtig	sehr wichtig	n=133	mw=3,6	md=4,0	s=1,1
keine Studiengebühren	überhaupt nicht wichtig	sehr wichtig	n=138	mw=3,3	md=4,0	s=1,4
Zuweisung durch Hochschulstart	überhaupt nicht wichtig	sehr wichtig	n=109	mw=2,9	md=3,0	s=1,6
Magdeburg ist keine Massenuniversität	überhaupt nicht wichtig	sehr wichtig	n=136	mw=3,7	md=4,0	s=1,1
großes Faktenwissen erwerben	trifft überhaupt nicht zu	trifft völlig zu	n=138	mw=4,5	md=5,0	s=0,7
Prüfungen bestehen	trifft überhaupt nicht zu	trifft völlig zu	n=142	mw=4,6	md=5,0	s=0,6
viel und intensiv arbeiten	trifft überhaupt nicht zu	trifft völlig zu	n=137	mw=4,2	md=4,0	s=0,7
sich mit theoretischen Fragen auseinandersetzen	trifft überhaupt nicht zu	trifft völlig zu	n=140	mw=4,1	md=4,0	s=0,8
Praxisbezug	trifft überhaupt nicht zu	trifft völlig zu	n=139	mw=4,6	md=5,0	s=0,7
wissenschaftlich arbeiten	trifft überhaupt nicht zu	trifft völlig zu	n=140	mw=4,1	md=4,0	s=0,8
Forschungsbezug	trifft überhaupt nicht zu	trifft völlig zu	n=137	mw=3,9	md=4,0	s=0,8
die Vereinbarkeit von Studium und Familie	überhaupt nicht wichtig	sehr wichtig	n=136	mw=3,6	md=4,0	s=1,2
die Vereinbarkeit von Studium und Nebenjob/Beruf	überhaupt nicht wichtig	sehr wichtig	n=138	mw=2,7	md=3,0	s=1,2
Möglichkeit eines Teilzeitstudiums	überhaupt nicht wichtig	sehr wichtig	n=137	mw=1,4	md=1,0	s=0,8
Studien- und Prüfungsordnung	gar nicht	sehr gut	n=139	mw=2,8	md=3,0	s=1,0
Möglichkeiten der Studienberatung	gar nicht	sehr gut	n=140	mw=3,2	md=3,0	s=0,9
Einzelheiten des BAföG	gar nicht	sehr gut	n=122	mw=2,4	md=2,0	s=1,1
Möglichkeiten für ein Studium im Ausland	gar nicht	sehr gut	n=134	mw=2,2	md=2,0	s=1,0
Arbeitsmarktsituation im angestrebten Tätigkeitsfeld	gar nicht	sehr gut	n=139	mw=3,5	md=4,0	s=1,0

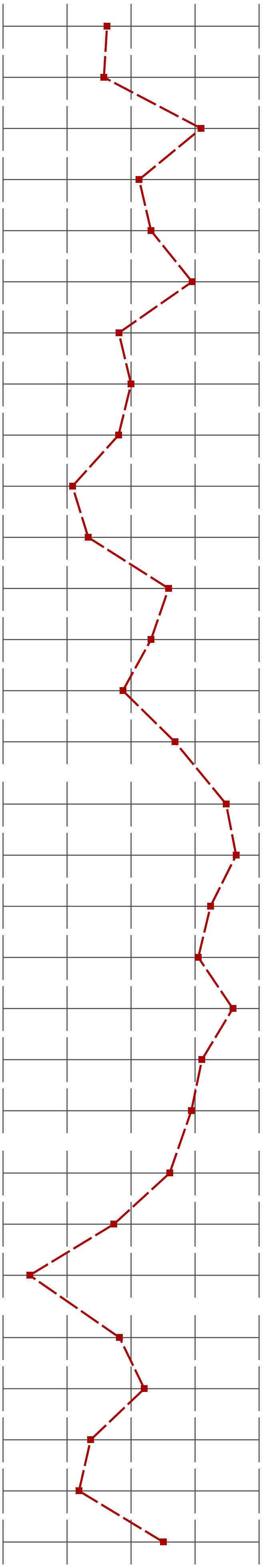

aktuelle politische Überlegungen der Hochschulentwicklung	gar nicht	sehr gut	n=140 mw=2,9 md=3,0 s=1,1
Abschluss des Studiums in der Regelstudienzeit	überhaupt nicht wichtig	sehr wichtig	n=138 mw=4,2 md=4,0 s=0,9
Bestehen des Studiums	überhaupt nicht wichtig	sehr wichtig	n=137 mw=5,0 md=5,0 s=0,2
den eigenen Maßstäben gerecht werden	überhaupt nicht wichtig	sehr wichtig	n=136 mw=4,5 md=5,0 s=0,6
einen Bereich finden der Spaß macht	überhaupt nicht wichtig	sehr wichtig	n=138 mw=4,7 md=5,0 s=0,5
Promotionsthema bearbeiten mit dem Ziel, einen Doktortitel zu erlangen	überhaupt nicht wichtig	sehr wichtig	n=136 mw=3,9 md=4,0 s=0,9
ein breites Allgemeinwissen aneignen	überhaupt nicht wichtig	sehr wichtig	n=139 mw=4,2 md=4,0 s=0,7
nachhaltiges Wissen erlangen	überhaupt nicht wichtig	sehr wichtig	n=138 mw=4,6 md=5,0 s=0,6
ein Leben" neben dem Studium "	überhaupt nicht wichtig	sehr wichtig	n=140 mw=4,3 md=5,0 s=0,8

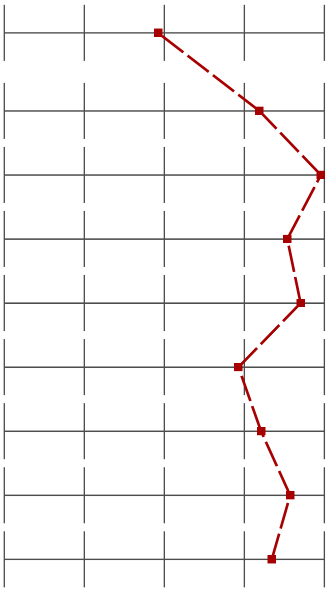

Auswertungsteil der offenen Fragen

1. In welchem Jahr haben Sie die Hochschulreife erworben?

5. In welchen Kursen haben Sie Ihre Hochschulreife abgelegt?

10. Was waren Ihre ersten drei Ortspräferenzen auf dem Zulassungsantrag der Quote, über die Sie zugelassen wurden?

14. Ist das Medizinstudium Ihr ursprüngliches Wunschfach?

17. Welche Informationsquellen haben Sie vor der Entscheidung für Magdeburg herangezogen?

25. Aus Ihrer heutigen Sicht, in welcher Fachdisziplin möchten Sie tätig sein (Einfachnennung)?

29. Jetzt würden wir gerne von Ihnen wissen, wo Sie sich selbst in 10 Jahren sehen.

31. In welchem Jahr wurden Sie geboren?

Matrikel-Nummer
